# Who Watches the “Watchman” and How Often?

**DOI:** 10.7759/cureus.10077

**Published:** 2020-08-27

**Authors:** Kyaw M Hlaing, Thein T Aung, Kevin Kravitz, Kamran Riaz

**Affiliations:** 1 Internal Medicine, LewisGale Medical Center, Salem, USA; 2 Cardiology, Miami Valley Hospital, Wright State University, Dayton, USA

**Keywords:** recurrent thrombosis, watchman device, left atrial appendage occlude, anticoagulation

## Abstract

Atrial fibrillation (AF) is a common cardiac arrhythmia that is encountered during the hospitalization. Sometimes, many patients cannot be anticoagulated to prevent AF-related cardiovascular accidents because of the risk of bleeding. In these cases, we recommend putting left atrial appendage (LAA) to prevent thrombus formation in the left atrium due to AF. There is no clear time frame of how long we need to follow up with echocardiogram to monitor device-related blood clot formation and continue anticoagulation therapy if there is recurrent thrombus formation after LAA placement.

We would like to present a case with AF in which the patient had epistaxis, which required to hold anticoagulation and arterial embolization. The patient agreed to the placement of the Watchman device and subsequently it was complicated by device-related thrombosis (DRT). The patient required prolonged anticoagulation treatment and follow-up echocardiogram to prevent DRT in the future.

## Introduction

Atrial fibrillation (AF) is the most common cardiac arrhythmia with a prevalence of six million patients in the United States alone [1]. Thromboembolism is the leading cause of mortality and morbidity in patients with AF. The left atrial appendage (LAA) is a source of intracardiac thrombus in more than 90% of patients who had stroke from AF [2]. Anticoagulation to prevent thromboembolism is the cornerstone of AF management. Many patients are not suitable to take long-term anticoagulation because of risk of bleeding or other contraindications. LAA occlusion strategies such as Watchman device (Boston Scientific, Marlborough, MA, USA) are approved for the prevention of stroke and systemic thromboembolism. Watchman device is a self-expandable nitinol cage deployed in the LAA using a transseptal approach. We would like to present a case of intracardiac thrombus formation on the Watchman device and management of anticoagulation in this specific scenario.

## Case presentation

A 62-year-old male with coronary artery disease (CAD), ischemic cardiomyopathy, reduced left ventricular function with ejection fraction of 15-20%, paroxysmal AF on anticoagulation with warfarin, and two prior cerebrovascular accidents (CVAs) presented with nose bleeding. His CHA2DS2-VASc score (congestive heart failure = 1 score, hypertension = 1 score, age ≥ 75 years = 2 scores, diabetes mellitus = 1 score, previous stroke/transient ischemic attack/thromboembolism = 2 scores, vascular disease = 1 score, age 65-74 years = 1 score, female gender = 1 score) was at least 3 (CVA and CAD) and because of the risk of thromboembolism, he was anticoagulated with warfarin. He had severe epistaxis requiring transfusions and nasal artery embolization. Due to heavy recurrent bleeding, he was unable to take long-term anticoagulation. The endocardial LAA occlusion by the Watchman device was offered as an alternative therapy. The patient agreed to the procedure, and the Watchman device was implanted without immediate complications such as perforation or pericardial effusion. Patient epistaxis, as a result of holding anticoagulant and embolization, was resolved.

**Figure 1:**
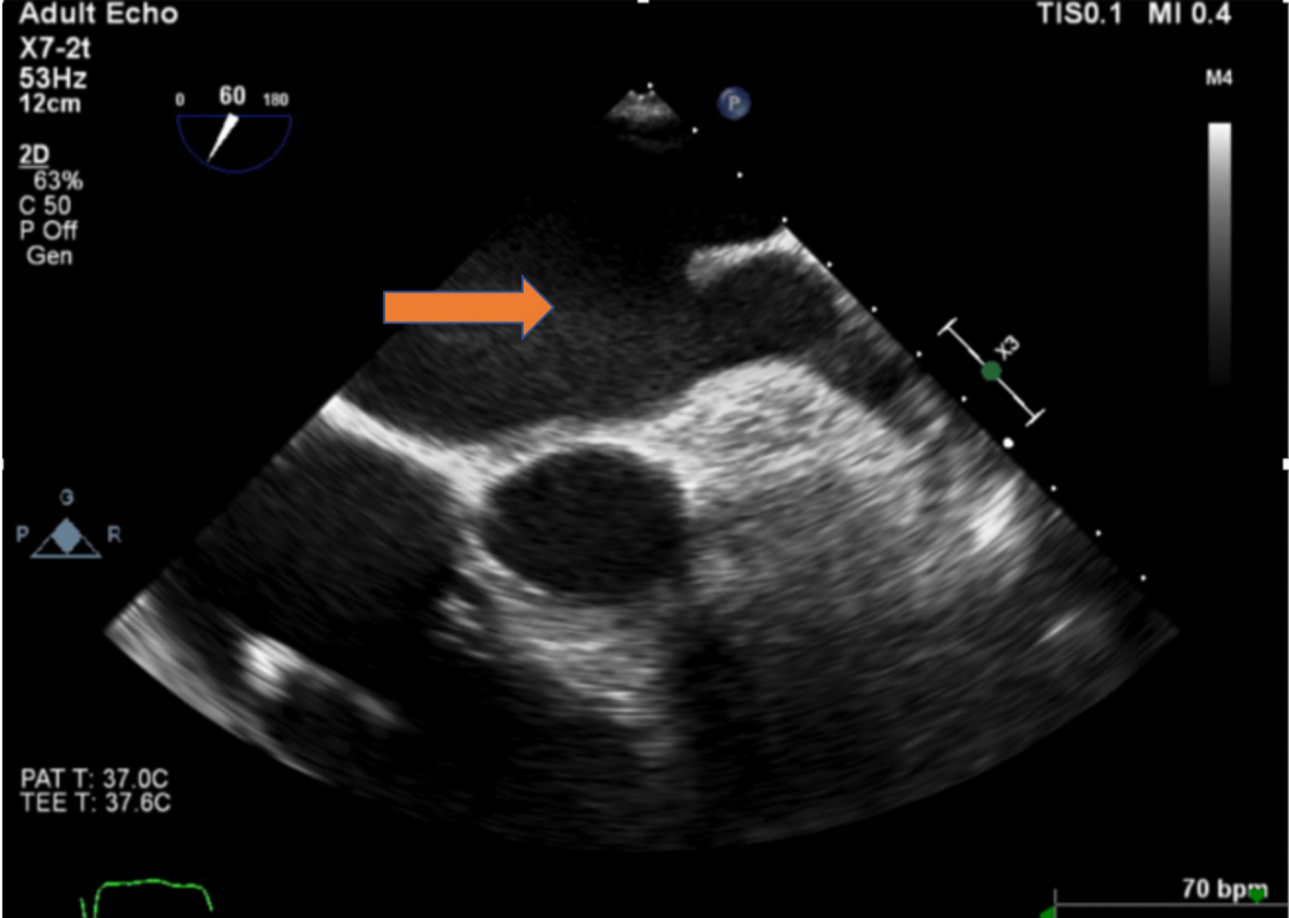
TEE in August 2016 showing left atrial appendage (red arrow) without thrombus. TEE, transesophageal echocardiogram

**Figure 2:**
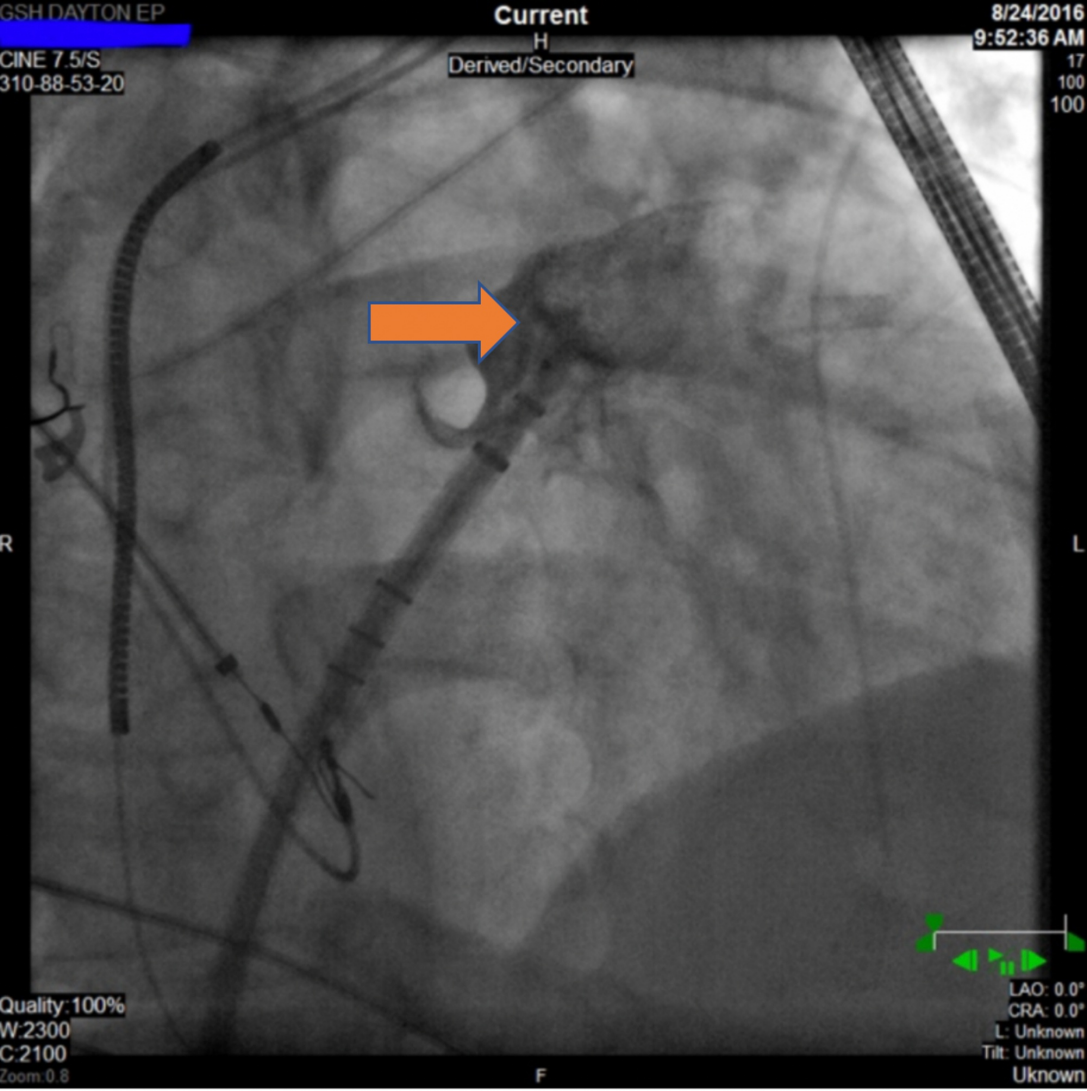
Fluoroscopic image while deploying the left atrial occlusion device Watchman (red arrow). TEE, transesophageal echocardiogram

**Figure 3:**
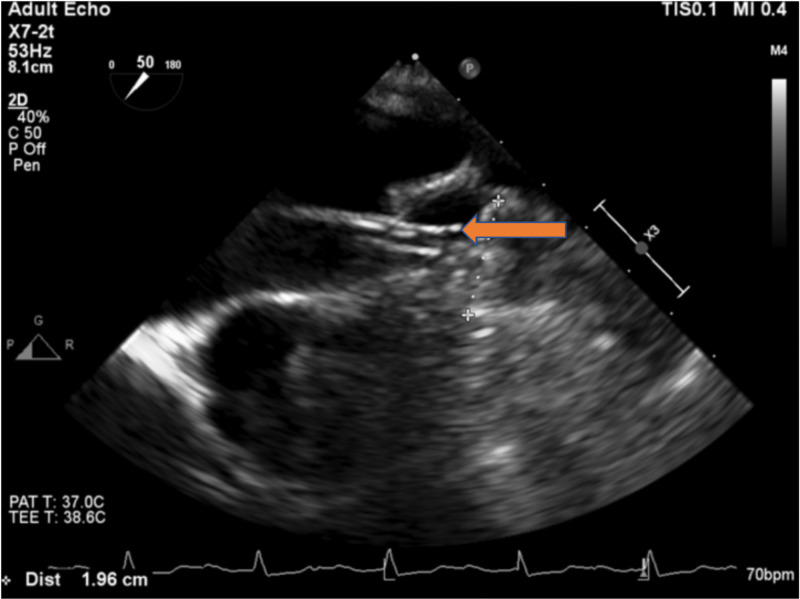
TEE showing a stable Watchman device without peri-device leak. TEE, transesophageal echocardiogram

Intraprocedure transesophageal echocardiogram (TEE) showed no intracardiac thrombus (Figure 1). There were lateral wall akinesia and severe global hypokinesis. The LAA diameter was 20 mm. The patient underwent a successful deployment of the 24-mm Watchman device (Figure 2). The subsequent figure (Figure 3) showed a stable Watchman device without peri-device leak.

As per the manufacturer guideline, anticoagulation with warfarin was prescribed for a total of six weeks after the device implanted. There was no history of interruption in anticoagulation with warfarin. However, the international normalized ratio (INR) level was found to be 1.5 (subtherapeutic) on follow-up clinic visit.

**Figure 4:**
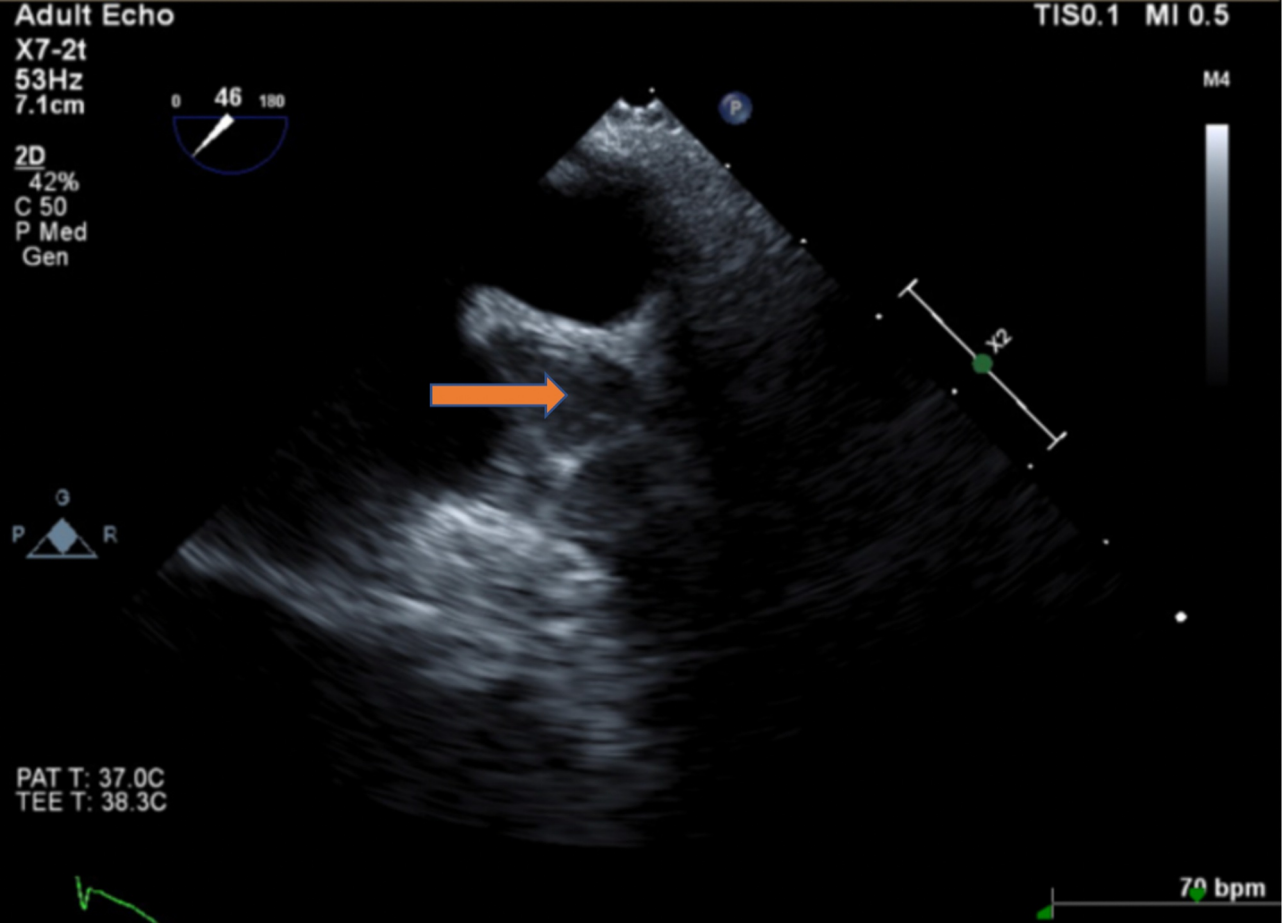
TEE six weeks after Watchman device deployment. Intracardiac thrombus (red arrow) was seen on top of the Watchman device. TEE, transesophageal echocardiogram

**Figure 5:**
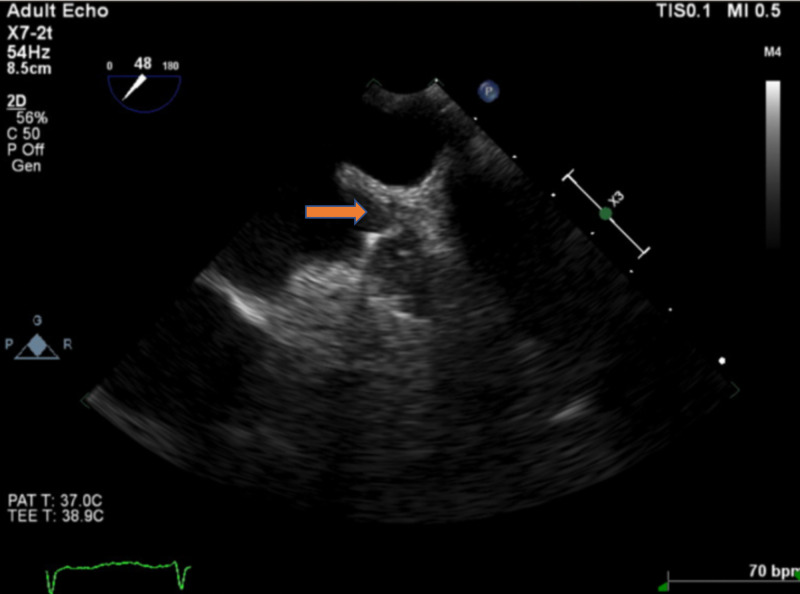
After three months of anticoagulation (4.5 months after Watchman device implantation), the left atrial thrombus was almost resolved (red arrow).

**Figure 6:**
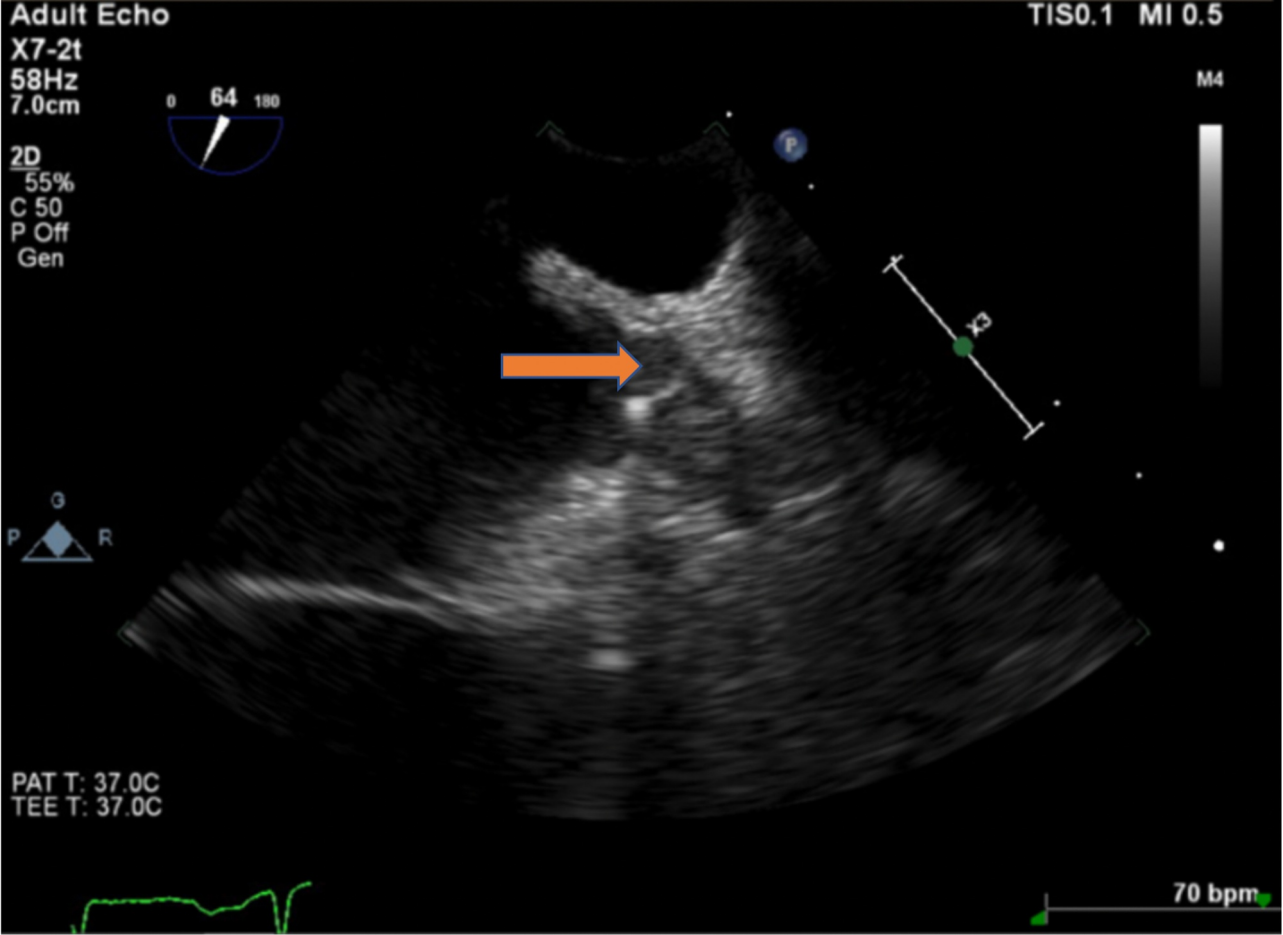
TEE six months after Watchman device implantation. The thrombus (red arrow) was grown back after cessation of anticoagulation. TEE, transesophageal echocardiogram

Follow-up TEE six weeks after the implantation showed stable device placement but with thrombus formation on the device (Figure 4). Warfarin therapy was extended for three more months. A repeat TEE after 4.5 months revealed resolution of the thrombus (Figure 5). Warfarin was stopped at that time, and he was switched to dual antiplatelet therapy with aspirin and clopidogrel. Follow-up TEE at six months showed recurrent thrombus formation layered on top of the Watchman device (Figure 6). He was reinstated on warfarin anticoagulation, with planned reevaluation showing the persistent thrombus. He was continued on oral anticoagulation with warfarin for the past year. The patient is still alive and well without any stroke.

## Discussion

The risk of thromboembolism in patients with AF is most commonly evaluated by the CHA2DS2-VASc score [3]. In patients with AF and high thromboembolic risk, anticoagulation treatment with warfarin or newer oral anticoagulants consistently improves mortality [4]. However, contraindications preclude many people from lifelong anticoagulation. Some patients are not suitable for long-term anticoagulation due to a variety of reasons such as (A) history of major bleeding, (B) inability to maintain a stable therapeutic INR or to comply with regular INR monitoring and unavailability of an approved alternative anticoagulation agent, or (C) either a medical condition, occupation, or lifestyle placing the patient at high risk of major bleeding due to trauma. The risk of bleeding can be also calculated by the HAS-BLED score, which includes hypertension, abnormal renal disease or liver function, stroke, history of bleeding, labile INR, elderly, and drugs with bleeding risk.

Patient compliance for long-term anticoagulation therapy was also consistently poor. A retrospective cohort consisted of more than 12,000 patients showed more than 40 % of patients discontinued anticoagulation within 120 days [5]. Bleeding is the most common reason for non-compliance.

LAA occlusion device provides an alternative therapy to prevent stroke and thromboembolism in patients with AF. The Watchman device is, by far, the most commonly implanted endocardial LAA occlusion device in the United States. The Watchman Left Atrial Appendage Closure Device for Embolic Protection in Patients with Atrial Fibrillation (PROTECT-AF) trial proved the safety and non-inferiority of LAA occlusion compared to the standard oral anticoagulation therapy [6]. The screening criterion was CHA2DS2-VASc score ≥ 1.

Post-procedural anticoagulation therapy includes warfarin for six weeks (45 days). The successful device implantation was confirmed by follow-up TEE at six weeks. If the device is stable and there is less than 5-mm peri-device leak, then the anticoagulation can be stopped. The patients were switched to dual antiplatelet therapy consisting of aspirin and clopidogrel from six weeks to six months. This protocol from PROTECT-AF trial was used as a guideline for Watchman device implantation.

Implanting a Watchman device comes with the procedural risk and complications. A recently published study, post-approval experience with Watchman device, reported a much lower procedural complication rate (1.28%) across six Watchman studies. The most common complication of the Watchman implantation procedure was pericardial tamponade, which was treated with pericardiocentesis. Procedure-related stroke was 0.18% [7].

Device-related thrombosis (DRT) is a severe complication after LAA occlusion device (Watchman) placement, with some cases necessitating surgical removal of the implanted device [8]. There are other case reports of Watchman device thrombosis that resolved with long-term anticoagulation. Wong et al. reported a case of DRT, which was successfully treated with short-term apixaban. They picked up the thrombus on the 30-mm Watchman device at six months follow-up, which was treated with three months of apixaban. Repeat TEE after three months showed complete resolution of DRT on the Watchman device [9].

The original PROTECT-AF trial reported DRT formation (5.7%), pericardial tamponade (4.3%), and procedure-related stroke (1.15%) [10]. A recently published meta-analysis by Dukkipati et al. covered four trials including 1,739 patients. The incidence of DRT formation was 3.74%. Stroke or thromboembolism was clearly higher in patients with DRT (7.46 vs 1.78 per 100 patient-years). The risk factors for DRT include prior history of stroke, permanent AF, vascular disease, LAA diameter, and left ventricular systolic function [11]. All the patients with DRT had their anticoagulation interrupted or subtherapeutic INR.

According to the list, our patient is at high risk due to prior history of stroke, and having CAD and left ventricular dysfunction with the ejection fraction of 20%. His LAA diameter was 20 mm and his AF was paroxysmal. Anticoagulation was not interrupted, but subtherapeutic INR became 1.5 immediately after the procedure.

Our patient had severe epistaxis, and his anticoagulation was interrupted for surgery shortly after the Watchman device implantation. Consequently, he developed intracardiac thrombus or DRT. We do not have guidelines or clinical data for the treatment of DRT. Previously published cases reported resolution of thrombus with short-term anticoagulation for three months. Anticoagulation with warfarin was restarted for three more months, and the thrombus resolved on the subsequent TEE. He was switched to dual anti-platelet therapy as the manufacturer recommended. However, the thrombus reoccurred at six months follow-up echocardiography after warfarin was stopped. Anticoagulation was restarted again to treat DRT.

Shunsuke et al. published a single-center statistic in August 2017 which showed a DRT of 3.4%. The authors noted that all patients with DRT discontinued either aspirin or warfarin. Treatment with aspirin and warfarin for six months resolved the thrombus on the subsequent TEE. They did not notice thrombus formation after six months [12]. DRT rate in a recently published meta-analysis which included four prospective FDA trials was reported as 3.74 % (65 patients out of 1,739) [13].

There is also a bright side to this gloomy problem. All the intracardiac thrombus will eventually be covered by endothelial cells and undergo endothelization at some point. An animal study by Schwartz et al. documented that the LAA occlusion device was covered at day 45 by endothelial cell migration. Complete endocardial lining coverage was seen at day 90 [14]. However, this study is conducted with animals and the findings are all autopsy findings. Currently, non-invasive imaging was not proven to document the full endothelization of DRT on the Watchman device. Therefore, our patient was continued on long-term anticoagulation with warfarin.

## Conclusions

LAA closure is currently offered as an alternative to oral anticoagulation in patients with contraindication for long-term anticoagulation. These patients are already exposed to high risk of bleeding. Overall, DRT risk of 3.7-5.7% is present. Risk factors for DRT include premature discontinuation/interruption of anticoagulation therapy, subtherapeutic INR, prior stroke history, permanent AF, LV dysfunction, and LAA diameter. DRT occurs only in patients who discontinued oral anticoagulation prematurely. 

Currently, FDA approved only for warfarin for 45 days and follow-up by dual antiplatelet therapy to six months before the device is completely endothelialized. No data are available for newer anticoagulation therapy post-device placement. 

There is a lack of guidance and data on how to manage DRT after Watchman device implantation. Most of the thrombus resolve with short-term anticoagulation. Current guidelines suggest six months of anticoagulation. The DRT in our patient resolved with short-term anticoagulation, but it came back after cessation of anticoagulation. We need more data to recommend the optimal treatment duration in patients with DRT. Meanwhile, we suggest longer warfarin duration and longer monitoring with TEE for those patients with subtherapeutic anticoagulation and DRT.
